# ‘We should be focusing on why we eat, what we eat and how it makes us feel, not how many calories it has’: a photovoice study exploring young people’s views on the out-of-home calorie labelling policy in England and their priorities for changing the local food environment

**DOI:** 10.1186/s12889-026-26716-7

**Published:** 2026-02-24

**Authors:** Vanessa Er, Camilla Forbes, Dalya Marks, Laura Cornelsen, Penny Breeze, Alexandra Kalbus, Cherry Law, Richard Smith, Kerry Ann Brown

**Affiliations:** 1 Faculty of Public Health & Policy, London School of Hygiene & Tropical Medicine, London, UK; 2 Faculty of Health & Life Sciences, University of Exeter, Exeter, UK; 3 School of Medicine and Population Health, University of Sheffield, Sheffield, UK; 4 Department of Agri-Food Economics and Marketing, University of Reading, Reading, UK; 5 Faculty of Epidemiology & Population Health, London School of Hygiene & Tropical Medicine, London, UK

**Keywords:** Food policy, Food labelling, Food environment, Food justice, Young people, Photovoice

## Abstract

**Background:**

Obesity is a complex problem, a perpetual challenge for governments to address. In 2022 as part of a government obesity strategy, a mandatory calorie labelling policy for out-of-home food sectors was implemented in England. Little is known about the impact of this policy on young people; therefore, this study explored young people views on the policy and their priorities for change in their local food environments.

**Methods:**

We conducted a participatory, qualitative study using Photovoice with 20 young people (12–17 years old) living in two communities in areas of high deprivation in England, a coastal town and an inner-city neighbourhood. Young people were involved in photography-led focus groups, a photo ‘walkabout’ in their local highstreets, and a local community photography exhibition. A textual-visual thematic analysis framework was used to analyse textual and visual data.

**Results:**

Young people described challenges with navigating complex out-of-home food environments and reflected on whether the policy was effective in addressing these challenges. Three themes were identified: (1) *Relevance of calories for informing healthy eating*. Few found calories alone helpful to gauge whether something was healthy. However, some expressed surprise at the energy content of drinks and found calorie information useful for understanding ‘liquid calories’. (2) *Commercial influences on food choices*. Participants were aware of how commercial activities shaped their food environments, often in unequal ways, and how targeted marketing/advertising for young people can be more influential than calorie information on menus and food labels. (3) *Value placed on the relational aspects of food.* Young people recognised food as more than fuel, valuing its social and cultural significance and believing it should be produced and sold ethically and sustainably.

**Conclusion:**

Young people viewed calorie labelling as unhelpful for making healthy eating choices, emphasising instead the need to address structural barriers and support for them to navigate increasingly complex and inequitable food systems. They demonstrated their role as informed food citizens by exercising social responsibility to shape their food environments. It is therefore vital to amplify their voices in public discourse and support their participation in shaping food policy and governance.

**Supplementary Information:**

The online version contains supplementary material available at 10.1186/s12889-026-26716-7.

## Introduction

Obesity is the most common form of malnutrition worldwide, with one in eight people identified as living with obesity in 2022 [1]. It increases risks of chronic non-communicable diseases, such as cardiovascular diseases, type 2 diabetes and certain cancers, all of which carry high health and wellbeing, economic, and societal costs [2]. In England, almost two-thirds of adults live with overweight or obesity, and one in five children live with obesity by the time they leave primary school, aged 11 years [3, 4]. These burdens are unequally distributed, with 29% of children aged 10–11 years living with obesity or being overweight in the most deprived areas of England, compared to 13% in the least deprived areas [3, 5]. This compounds diet-related health inequalities across the life-course, as childhood and adolescent obesity can be a predictor of ill-health later in life [6].

The out-of-home (OOH) food environment in the UK is a particular area of concern for tackling obesity rates. Food consumed OOH represents 15–39% of food expenditure in England [7] and accounts for 12% of total energy intake in the UK between 2019 and 2023 [8]. The number of restaurants and takeaway outlets in the UK has increased over time [9], and follows a socially-patterned distribution, with higher densities of OOH food outlets concentrated in more deprived areas [10]. Furthermore, OOH foods available in more deprived areas are less healthy than those in more affluent areas [11], further exacerbating diet-related health inequalities.

### Out-of-home calorie labelling policy

Governments around the world have taken various structural and individual behaviour change interventions to address the obesity epidemic. One such intervention, which has been implemented in different forms in several countries, is to display calorie information (energy from food, kcal/kJ) on menus or at the point of food purchases in OOH settings [12]. In April 2022, a mandatory OOH calorie labelling policy was introduced in England, which requires large businesses (over 250 employees) selling food and drinks for immediate consumption (e.g., in high street or online versions of restaurants, cafes, fast food chains) to display calorie information on menus or alongside the foods for sale [13].

The OOH calorie labelling policy aims to support behaviour change by helping individuals make healthier food choices. It can also facilitate change in the food environment by encouraging businesses to reformulate the foods or meals available by reducing their energy content [13]. Evidence on its effectiveness, however, is mixed. A Cochrane review, which predominantly included experimental studies conducted in the USA, and included all calorie labelling (menu, alongside product, and on product labels), reported a small reduction in calories selected and purchased, with weaker evidence on its effect on consumption [14]. Audit and simulation studies have also suggested that calorie labelling policies can drive reformulation with potential cost savings and health benefits [15–18]. However, UK studies evaluating the policy in real-world settings consistently found no evidence of change in calories purchased or consumed [19–21].

### Young people’s involvement in shaping food policy

Young people are a key demographic of OOH food consumption. Approximately 80% of 11 to 18-year-olds in the UK consumed food or drink OOH between 2019 and 2023 [8]. There is consistent evidence that adolescents and younger people are more likely to purchase OOH foods and derive a higher proportion of their dietary intake (e.g., energy intakes) from foods in this sector [22, 23].

The degree to which the UK’s calorie labelling policy considers its relevance to young people is unclear, and there has been limited involvement of young people in its evaluation. Existing evidence has largely focused on assessing its effectiveness [14, 19, 21], while qualitative studies have primarily captured the perspectives of adults and businesses [24–27]. Youth engagement in public health policy remains scarce, with little research exploring their views, particularly regarding policy implementation and evaluation [28]. This represents a significant gap, as tackling complex public health challenges such as obesity requires participatory approaches that incorporate diverse perspectives to be effective [29]. Young people have demonstrated their desire to play an active role in shaping their local food environment, and they have a right to influence policy decisions that affect their lives [30, 31]. Accordingly, the current study aimed to explore young people’s views on the calorie labelling policy and understand their priorities for change in local food environments.

## Methods

### Reflexivity

Our existing understandings of health equity, food systems, and youth participation in policymaking shaped the research process. As public health researchers, we situate our work within the broader determinants of health, which we consider fundamental to both explaining and addressing health inequalities. We approach public health challenges, including diet-related problems, such as obesity, through a systems lens, recognising the complex web of interdependent factors that shape health outcomes. Accordingly, the calorie labelling policy is understood as one of several approaches for addressing obesity within a complex food environment. While recognising its potential role in informing healthy food choices, we were also cognizant of its limited effectiveness and potential unintended consequences for young people. This perspective informed the study design by broadening the research questions to capture young people’s views of their local food environment, including a walkabout in their local high streets, and by explicitly exploring potential unintended consequences such as unhealthy food restriction. We remained mindful of our perspective when facilitating group discussions to avoid steering participants toward our own views. For example, we did not correct participants’ understandings of calories, calorie sources, or recommended daily intake, and we structured discussions to begin with open-ended questions about the policy overall, deliberately probing for both perceived positives and negatives.

We also believe that everyone, including young people, has the right to good health and to have their voices heard, consistent with Article 12 of the United Nations Convention on the Rights of the Child [32]. At the same time, we acknowledge the inherent power imbalances within research processes, particularly given our relative privilege and authority as adults. To mitigate these imbalances, we co-produced the study with a Young Persons’ Advisory Group (see Public Involvement and Engagement) and used Photovoice, a participatory research approach designed to centre young people’s perspectives. This approach gave young people agency and autonomy over which photographs they took and selected for group discussions and the photo exhibition, with discussion topics emerging from participants’ photo choices and shaped by peer interactions. Engaging in reflexive practice through writing and reviewing field notes, team discussions, and working with a young advisory group also challenged our assumptions about what young people notice and care about regarding food systems. We had not expected participants to explicitly raise social, environmental, and ethical concerns, including labour conditions, food waste, and animal welfare. Their insights prompted us to pay greater attention to these aspects in the interpretation and framing of the findings and underscoring the importance of supporting young people as active agents in shaping their food environments and policies.

### Study design

We conducted a participatory, qualitative study using Photovoice [33], which we adapted for this study. Participatory research approaches centre the views and experiences of participants, emphasising the co-creation of knowledge between participants and researchers [34–37]. These approaches can benefit participants through action, advocacy, or policy change, for example, by building collective capacity to address health inequalities [35, 36].

Photovoice is a visual participatory research method that has been widely used in health research with children and young people [38–40]. It enables individuals, particularly those from disadvantaged or marginalised communities, to record and reflect on the strengths and concerns of their communities. Visual images enable deeper reflection on lived experience than traditional methods such as interviews [33],, and give participants control over the stories they tell, which may be difficult to convey in words alone [33]. Furthermore, group discussions of photographs foster critical dialogue and collaborative learning, with researchers acting as facilitators, making the research process itself as meaningful as the outcomes [41].

Photovoice typically culminates in a photo exhibition that promotes dialogue between participants, their communities, and stakeholders, making it an effective tool for amplifying youth voices in policymaking [35, 36]. The study is reported in accordance with COREQ guidelines to ensure transparency [41–43].

### Public involvement and engagement

This study was co-produced with a Young Persons’ Advisory Group (YPAG) comprising eight students aged 14–16 years from a coastal secondary school located in an area of high deprivation. The group acted as advisors to the project and was distinct from the young people who participated in the research activities. The YPAG played an integral role in the study, contributing to the development of participant information leaflets and consent forms, as well as the design of the *Photovoice* session format and topic guide questions. Their input changed the wording of documents and the format of sessions, making them more accessible to participants. They were also involved in the data analysis process by sense-checking the initial themes and providing reflections on the findings, which added to the development of the presented themes. The group shared their insights and experiences as advisors during the final project dissemination event. To acknowledge their expertise and time commitment, YPAG members received monetary vouchers as remuneration.

### Participant recruitment

We purposively sampled participants based on age (12–18 years), geographical location (coastal/inner-city settings in England), and area-level deprivation (within the top third most deprived areas of the UK, based on 2019 English indices of deprivation [44]). The inclusion of both coastal and inner-city settings aimed to capture how the policy is perceived and experienced in areas characterised by high levels of deprivation and diet-related health inequalities. We did not sample by gender or ethnicity as these factors were not pertinent to the study objectives. Participants were recruited through two community partner organisations that work with young people, with whom the researchers had an existing relationship. These organisations hosted the sessions in their community spaces, provided ongoing support throughout the study, including the dissemination of study findings. Participants and their parent/carers were provided with information about the study, and both were asked to provide written consent. Additional consent was sought for the use of participants’ photographs for research and educational purposes (Appendix 2). The final sample comprised 20 young people aged 12–17 years, including 11 from the coastal setting and 9 from the inner-city setting. In the coastal group, there were four girls and seven boys, and attendance was consistently high, with a minimum of eight participants present at each session. In the inner-city group, there were seven girls and two boys; all participants attended every session except for two who were absent from the final session.

### Data collection

We consulted community partners to design data collection around young people’s schedules, resulting in different formats across sites. In the coastal area, five weekly 1.5-hour sessions were held after school between May and July 2024. In the inner-city area, sessions were conducted over fewer but longer periods (three consecutive days, each lasting up to four hours) during school holidays, with two separate groups recruited in May and July 2024.

Across both sites, sessions followed a common protocol, guided by session plans and a topic guide (Appendix 3) to ensure consistency. Participants were introduced to the study, received photography and ethics training (Appendix 3), discussed their awareness and understanding of the calorie labelling policy, and took part in an accompanied “walkabout”. The route at each site was determined through discussions with the groups, focusing on where they socialised and/or areas with a high density of OOH food outlets near their homes. This enabled participants to photograph their local food environments while allowing researchers to observe their interactions with calorie labelling. Given the participants’ age and advice from our community partners, researchers accompanied the ‘walkabout,’ which also facilitated informal, conversations. Participants were provided with a study camera and given an opportunity to practice taking photographs as part of the photography training.

Following the walkabout, the researchers downloaded the photos onto a study laptop, which were then printed or projected for group discussions where each participant reviewed and selected photographs they wished to discuss. Mindful of the potential sensitivities surrounding calorie counting and disordered eating, the research team allowed space for these issues to be raised and guided conversations sensitively when they arose. We encouraged all participants to contribute, to discuss both their own and others’ photographs, and worked to create a safe and trusted environment throughout the sessions. These discussions were recorded, transcribed by an approved transcription service that complies with UK Data Protection laws, and anonymised. Reflective fieldnotes were written after each session, including the ‘walkabout’. Participants received vouchers at the end of each session in recognition of their time and contribution.

Each participant then selected two or three of their own photographs and developed titles and captions to communicate their views within the exhibition. Photo exhibitions were held at both sites, attended by the young people, their families, local stakeholders, and community members, and included a Q&A session facilitated by the research team.

### Data analysis

Data included photographs and captions selected for the photography exhibitions, additional photographs discussed (but not exhibited) during focus group sessions, focus group transcripts, and researcher fieldnotes. All data were uploaded to and analysed using NVivo 14 software.

Our analysis was guided by the Textual-Visual Thematic Analysis Framework [45]. First, we conducted thematic analysis of all textual data, drawing on Braun and Clarke’s approach [46] to coding. We analysed data deductively by applying an initial coding framework informed by the study objectives and underpinned by a wider determinants of health perspective. At the same time, we coded data inductively to capture findings that did not fit with the framework. CF and VE completed initial coding independently for each site, with DM and KAB double-coding 10% of the data to sense check the coding framework, which was iteratively refined throughout the analysis.

Next, CF and VE analysed visual data alongside textual data by following three key principles: (a) respecting participants’ interpretations through captions and focus group transcripts, (b) focusing on visual aspects relevant to the research questions, and (c) examining whether visual and textual data overlapped, complemented, contradicted, or diverged from one another [45].

The final stage involved considering the dataset as a whole and reporting findings on the relationships between focus group transcripts, photographs, and captions. Mind maps were used to organise codes, and regular team meetings facilitated the identification and conceptualisation of themes to ensure they captured the central meanings and patterns in the data. The YPAG also contributed to analysis by sorting photographs and captions into themes and creating ‘blackout’ poems (erasing words from text to form a poem) to highlight salient points and sense-check initial themes.

## Results

Three main themes were identified: (1) the relevance of calories for informing healthy eating, (2) commercial influences on food choice, and (3) the value placed on the relational aspects of eating. A common thread across all three themes was the interconnected challenges young people faced in navigating complex out-of-home (OOH) food environments, alongside a generally negative perception of the calorie labelling policy.

The results for each theme are illustrated using verbatim focus group transcripts, a selection of photographs and captions displayed in the photography exhibitions, and additional photographs that elicited significant discussion during sessions. Quotes, photographs, and captions are labelled by study site (Coastal/Inner-city) to maintain participant confidentiality, and any identifiable faces have been blurred. Exhibition discussions were not recorded; summaries of these events are reported elsewhere [47, 48].

### Relevance of calories for informing healthy eating

Overall, young people did not find calorie labelling helpful in assessing whether food products were healthier or less healthy, which was a key consideration for them. While calorie labelling was seen as useful in some contexts, it was largely regarded as irrelevant or potentially harmful, as it drew attention to calories in isolation from wider healthy eating messages. They described various sources of nutritional information, noting how calories are portrayed differently, sometimes as a source of energy, and other times as fattening. They generally wanted more comprehensive information, such as nutrient content, rather than calories alone.

#### “It doesn’t really tell me anything”

**Fig. 1:**
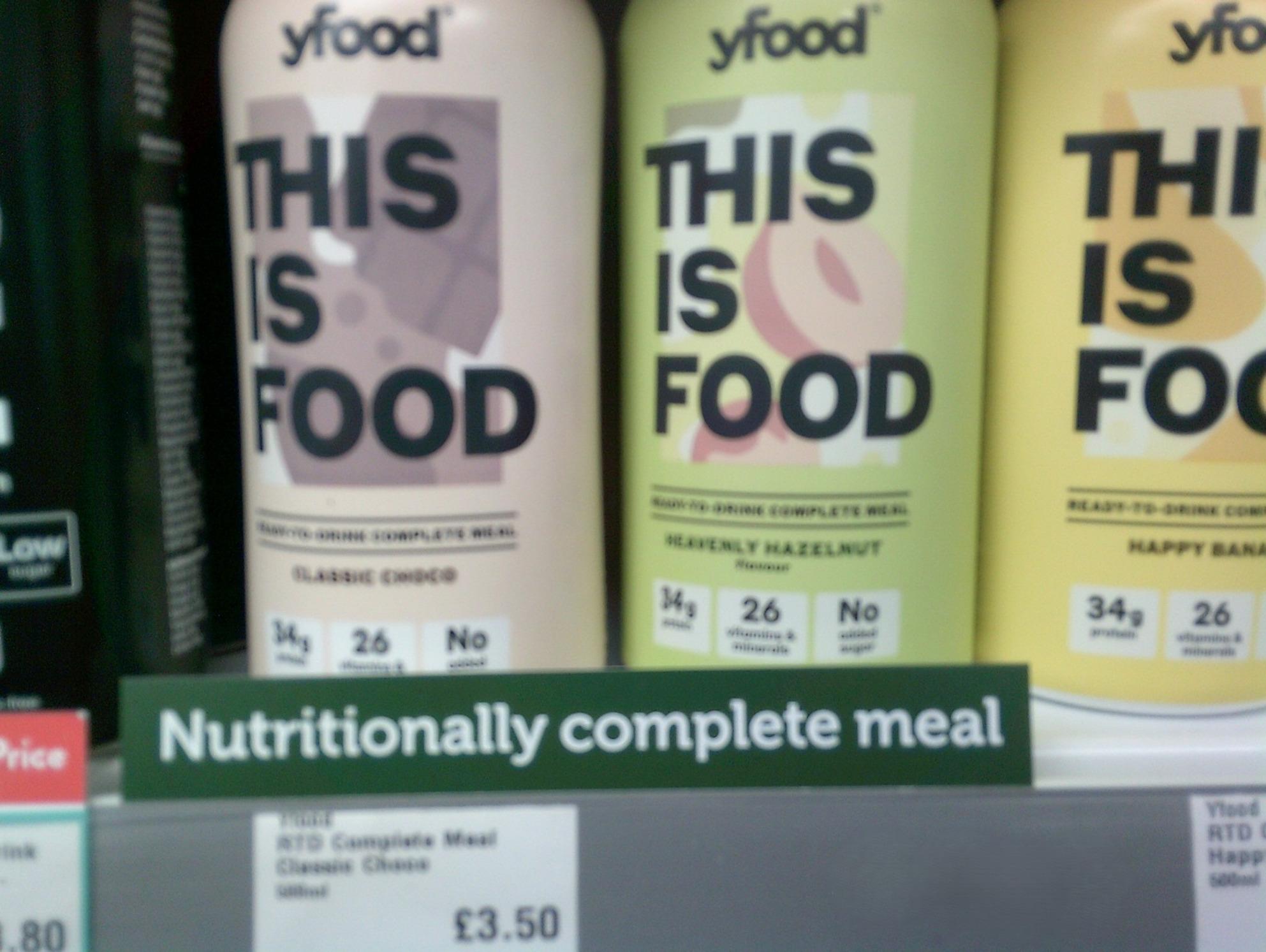
Food is more than fuel. ‘When I look at this photo my body fills with rage, it feels like all the work we have put into solving diet culture and negative relationships with food back dates and we are back to habits of labelling foods ‘good’ and ‘bad’ and trying so desperately to find food substitutes instead of tackling the bigger issue the world has with food…We eat for two reasons 1) satisfaction or 2) nutrition and sometimes both, and that’s ok. We should be focusing on why we eat, what we eat and how it makes us feel not how many calories it has. So, this photo for me captures how toxic and harmful calorie and labelling can be and how we need to change it for the better.’ (Coastal exhibition)

Young people largely perceived the display of calories on menus as irrelevant or even unhelpful in supporting them to make healthy eating choices. They viewed the policy as a distraction from addressing the structural barriers to healthy eating and suggested it could undo efforts to promote a healthy relationship with food, doing little to challenge harmful diet culture. They expressed concern that an excessive focus on calories, particularly displaying calorie information prominently, could cause distress or lead to unhealthy, restrictive eating behaviours (Fig. 1).

*‘It should be an option to be seen if you do or don’t want it*,* but it shouldn’t be out in the open. It should be something that you can find*,* but you should only find if you’re actively looking for it*,* not something in big*,* bold letters that catches your eye before the name of the food does. I think it’s important that it’s there*,* but it’s not going to be something someone who’s paranoid about eating can notice to stop them from eating. There’s this balance between it needs to be there*,* but it can’t be there too obviously*,* or it could cause people with eating disorders to stop eating certain foods. Findable*,* but not bold.’ (Coastal)*.

However, some young people considered the calorie labelling policy to be useful for highlighting the calories associated with drinks. Many young people were unaware of the high energy content of commonly consumed beverages, especially sugar-sweetened milk and coffee drinks. In this context, they felt calorie labelling helped raise their awareness and understanding of what they were consuming.

*‘… I would think of calories is eating calories*,* I don’t really think about drinking calories*,* which is like*,* I think some people don’t think that as well*,* because I would get a drink and I’d be like*,* OK*,* it’s cookies and cream*,* like I understand that’s not very healthy. But I wouldn’t think it had*,* I wouldn’t think it would have more*,* like*,* calories than a cheeseburger.’ (Inner-city)*.

**Fig. 2:**
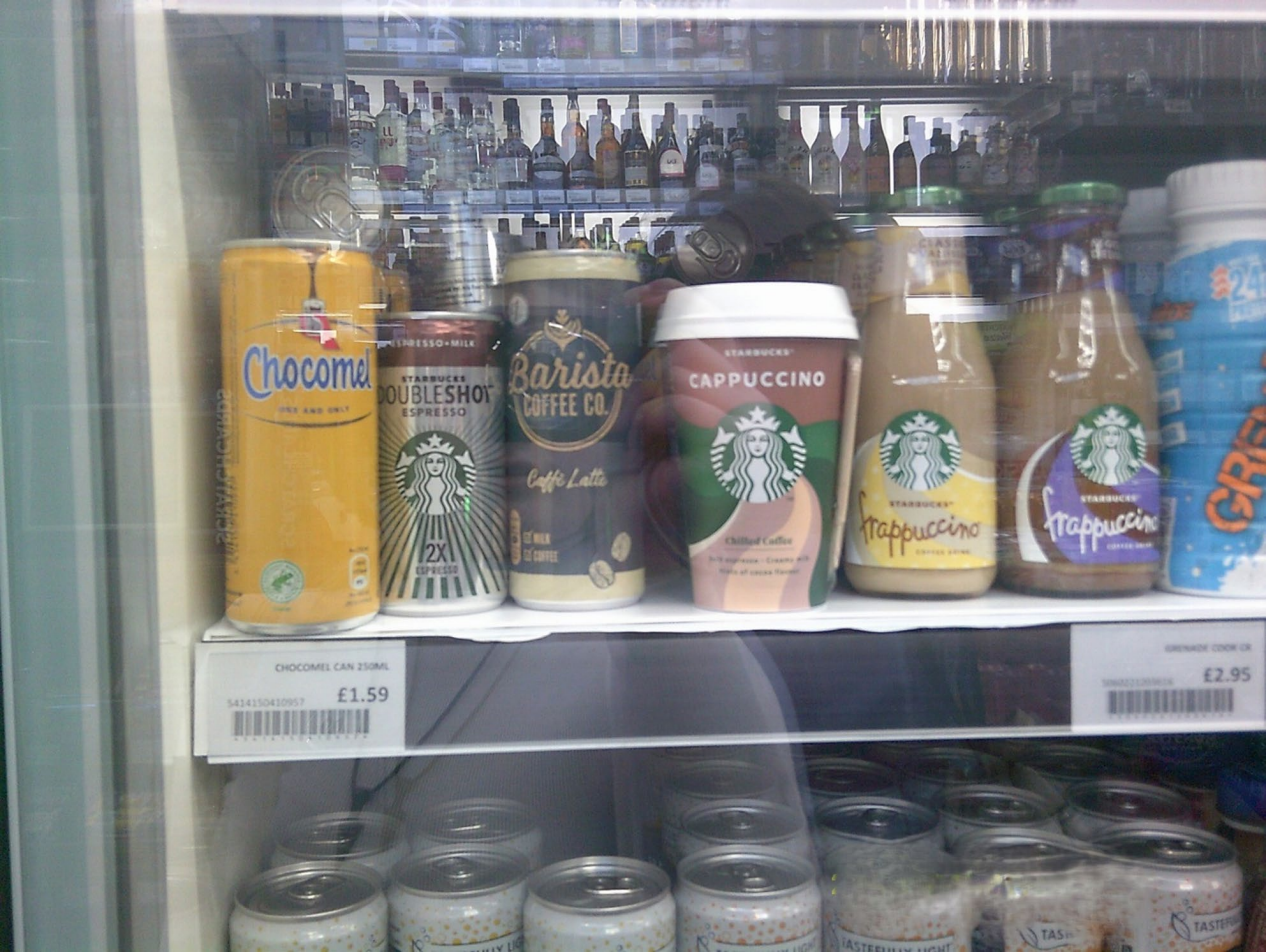
Liquid calories. The photograph elicited discussion, yet was not selected by participants for the exhibition, hence no caption (Coastal discussion group)

*‘…Starbucks… they brand themselves as being sweet coffee*,* which*,* you know*,* sweet coffee doesn’t exactly brand itself as healthy*,* yet somehow*,* they’re thought as healthy*,* because coffee can’t be sugary.’ (Coastal*,* discussion group referring to* Fig. 2*).*

**Fig. 3:**
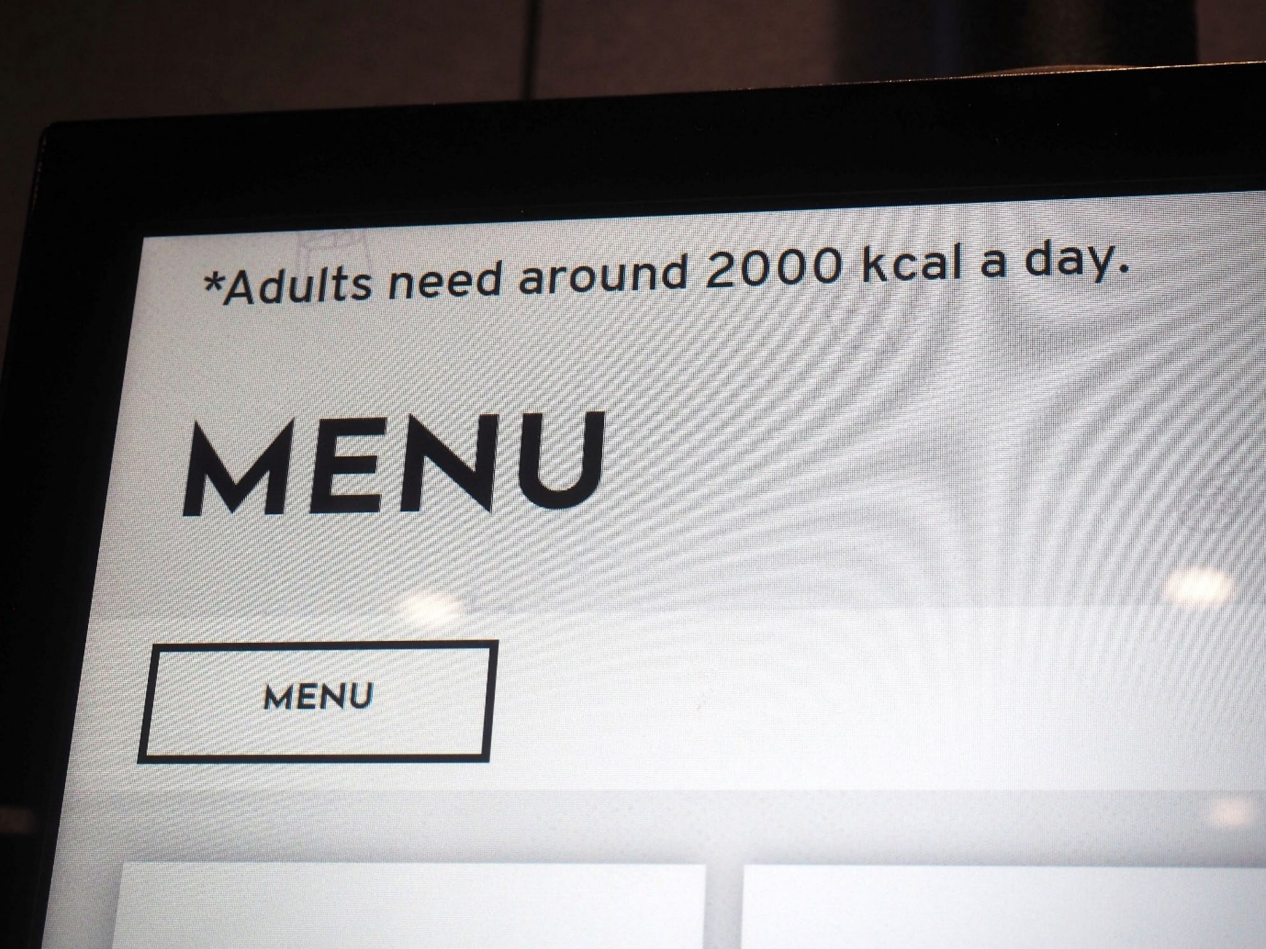
2000 Calories. ‘This image is powerful because it gives you the facts behind the appealing image. The reality is that one burger adds up to all the calories a kid should be eating in a whole day. It’s good to know how much calories you should have, but I think it kind of should be saying, how much calories kids should have, because it’s just adults and it’s different for kids. (Inner-city exhibition)

Similarly, some young people found the statement “adults need around 2,000 kcal a day,” which businesses are required to display under the policy, useful for gauging whether someone had eaten too much. However, they felt it was not personally relevant, as it is targeted at adults, leaving them unsure how it should be interpreted by young people (Fig. 3).

#### Accessible, useful, and relevant nutritional information needs

Overall, there was a strong and widely shared sentiment among young people that to help them navigate food environments and make healthier choices, they needed more information than the total number of calories. They preferred to know the ingredients or nutrient content of foods in addition to energy content. Participants also discussed other food labelling schemes, such as the UK traffic light labelling scheme, which uses red, amber, and green colours to indicate levels of total fat, salt, free sugars, and energy content. Many felt this scheme was more useful for quickly gauging the ‘healthiness’ of a food item compared with calories alone, although some still considered the scheme over-simplistic, as it encouraged viewing food as either good or bad.

*‘I think they’re unnecessary [calories]*,* I don’t know. I think the ingredients and how much of everything is really important*,* but I feel like there’s too much of a pressure on calories and not enough on like*,* the actual nutrients’ (Coastal)*.

Young people found it confusing to navigate the sources of nutritional information, as they encountered contradictory and mixed messages that made it difficult to know what was useful or relevant to them. Schools and social media were two key sources of information. The participants reported that the nutritional education they received in schools, which was framed around the Eatwell plate, was minimal, unmemorable, and largely irrelevant to the information they sought as they started making autonomous food choices. They recognised, however, that calories are portrayed more positively at school, as a source of energy derived from food, which contrasts with media narratives that typically associate calories with weight gain and other negative connotations.

*‘The Eatwell plate and it… like*,* it says like*,* these foods are good for you because these foods do this and these foods*,* like you can enjoy them but like*,* be mindful when you eat them. But I feel like that all goes out the window and when you like*,* grow up*,* you don’t know everything…’ (Coastal)*.

*‘But*,* as [name of participant] said*,* calories is just energy*,* so it does contribute to your weight*,* but at the same time it can be*,* the way that it’s portrayed in media and just like as it’s not taught in classrooms it’s just like calories it’s fat*,* like you have to watch your calories if you eat and it’s more about like what you’re eating.’ (Inner-city)*.

Social media platforms, such as TikTok, Instagram, fitness apps, Snapchat and YouTube, were described as dominant sources of information. Most had viewed nutritional advice on these platforms, even if it was not actively sought (e.g., shared by friends or automatically recommended by the platforms’ algorithms). They believed it was easier to learn from social media platforms than in school, although they recognised a need to ‘fact check’ information presented on social media, which was not necessarily perceived as trustworthy or accurate.

“*It feels easier to learn on TikTok*,* but you always have to actually fact check it… but it’s educational and especially since they made the videos*,* they can be longer now*,* so you can have more in depth information*,* but also*,* it’s not the most reliable source” (Inner-city).*

### Commercial influence on food choices

Young people’s accounts revealed that commercial entities and activities shape their OOH food environment and strongly influence their food choices. They perceived the commercial practices of some large food corporations as limiting their ability to act on their knowledge and intentions of eating a healthier diet in two ways. First, large food corporations shape access to food, often in unequal ways, by producing and selling cheaper and less healthy foods. Second, they invest substantial resources in advertising and marketing foods high in fat, salt, and sugar, frequently targeting children and using misleading tactics.

#### Unfair access to healthy foods

Young people were cognisant of inequality in access to food. They highlighted how families on lower incomes do not have fair access to healthy foods, making it harder for some households than others to have healthy diets. These challenges were seen as especially pronounced in areas of high deprivation and they drew attention to the broader health inequalities experienced by those living in more disadvantaged communities.

*‘Low income communities have way more [big chains]*,* I guess because of their environment as well*,* have worse health*,* but it’s also*,* like a little*,* a loaf of bread is way cheaper than a proper good wholemeal or something*,* it’s just*,* because you can just buy it so easily*,* or a pack of bagels and I don’t know*,* just a packet of some sort of fake sandwich meat.’ (Inner-city)*.

Young people associated large food corporations with selling ‘junk’ food (energy-dense, nutrient-poor, and ultra-processed food), typically produced and sold at low prices to maximise profit. In contrast, local independent businesses near schools, such as hot food takeaways or corner shops that they frequented, which also sold energy-dense and nutrient-poor food at low prices, were not subject to the same level of scrutiny. Despite expressing a preference for healthier, more diverse, and local food options, cost remained a primary factor influencing their OOH food choices, leading them to continue frequenting large chains and less healthy outlets.

*‘I mean*,* it is just like cheap [popular burger chain] and like it tastes nice and stuff. And when I’m out with my friends*,* I’m not trying to spend like £25 on a meal.’ (Inner-city)*.

*‘like there’s this chicken place like right next to my school*,* and it’s discounted after school time…It’s like*,* if you go after school then it’s*,* urm*,* £1. But if you go like on the weekend*,* then it’s like £2.’ (Inner-city)*.

They believed businesses and governments could make healthier food options more affordable and accessible, for example through subsidies, and that this would be more impactful than the calorie labelling policy.

*‘It’s like*,* the government could do more to*,* like*,* make sure that people with*,* like*,* smaller budgets can actually*,* like*,* eat healthier. There’s a lot of*,* like*,* fast*,* like*,* or like*,* ready meals are very*,* like*,* cheap and accessible…’ (Coastal)*.

*‘Why would you promote being healthy when you know people can’t afford it so then they will fall back on stuff that is not healthy for them*,* will damage their health*,* give them all sorts of skin problems*,* like health problems*,* more lasting health problems*,* why would you do that to a community and especially for young people as well*,* it doesn’t*,* just doesn’t make sense for me.’ (Inner-city)*.

#### Constant exposure to advertising and marketing

A common concern among young people was the constant exposure to food advertising and marketing, both in their physical surroundings and online. They expressed frustration at the pervasive advertising and marketing that entice them to buy foods high in calories and nutrients of concern (total fat, sodium, and free sugars), which made it harder for them to make healthier food choices.

*‘Yeah*,* totally. I mean*,* just going out there*,* we… I mean*,* just how everything’s quite… I mean*,* so sweet*,* it’s all quite pink and fluffy and*,* like*,* there’s a lot of*,* like*,* “hey*,* look*,* this is all fun and rainbows.” It’s going to give you bad teeth and get you fat and it’s like*,* I think*,* dialling down on some of that part of it.’ (Coastal)*.

**Fig. 4:**
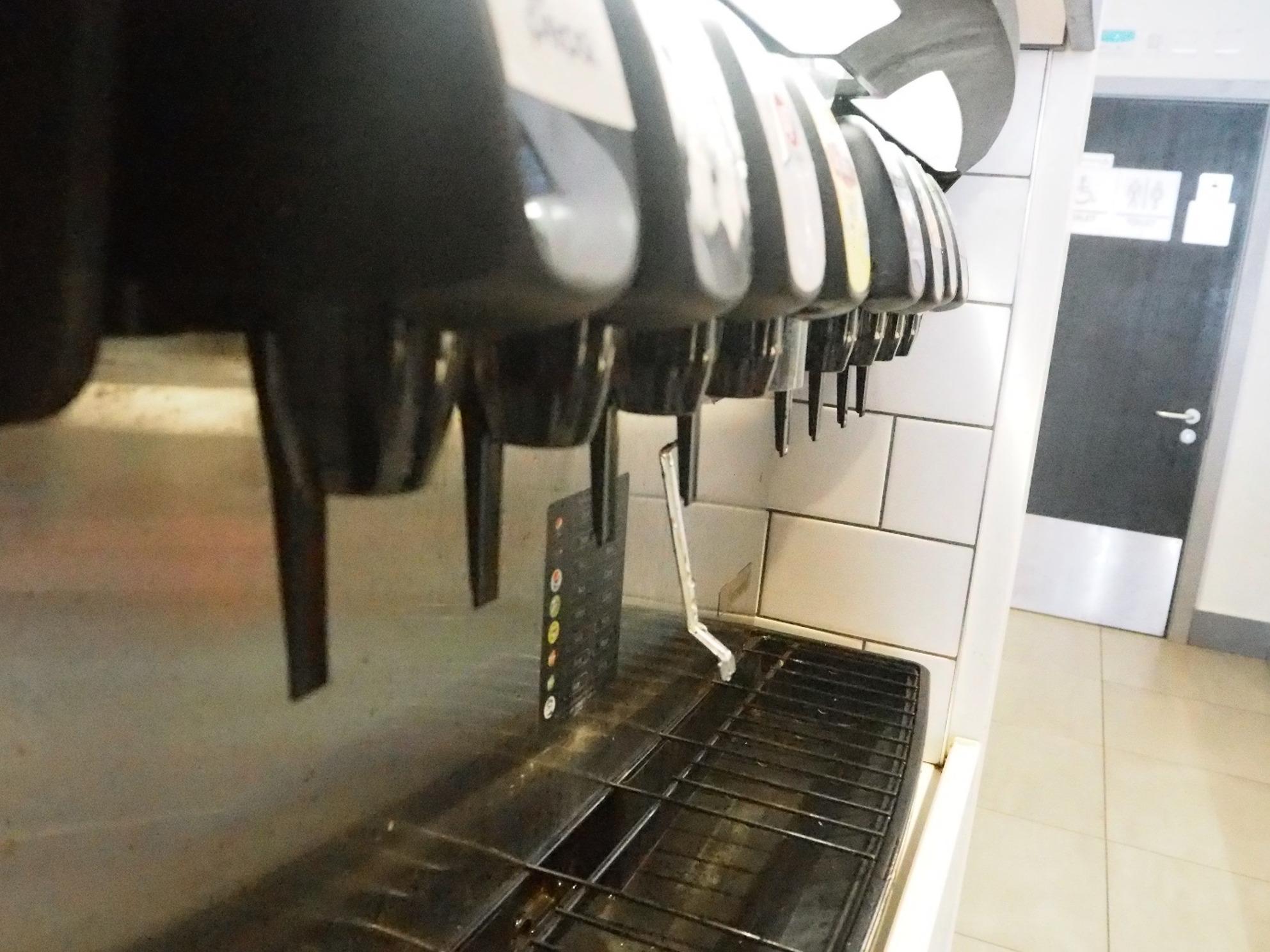
Society makes us fat. ‘The people in charge of making and selling the food, policies around food and stores we buy it in is something that makes people vulnerable and insecure about what we eat. Things like discounts, special offers are all around us.​ All around us we see signs that advertise “all we can eat” and bottomless drinks makes it hard for us to say no to more food because it is there and it is free. This makes us eat more than we should or want to or need.’ (Inner-city exhibition)

They identified various tactics used by food corporations, such as leveraging celebrities and influencers through social media to promote their products, offering promotions that encourage customers to purchase larger portions at lower prices, targeting children and young people through prize draws and games, and using bold colours and popular characters in advertising (Fig. 4).

**Fig. 5:**
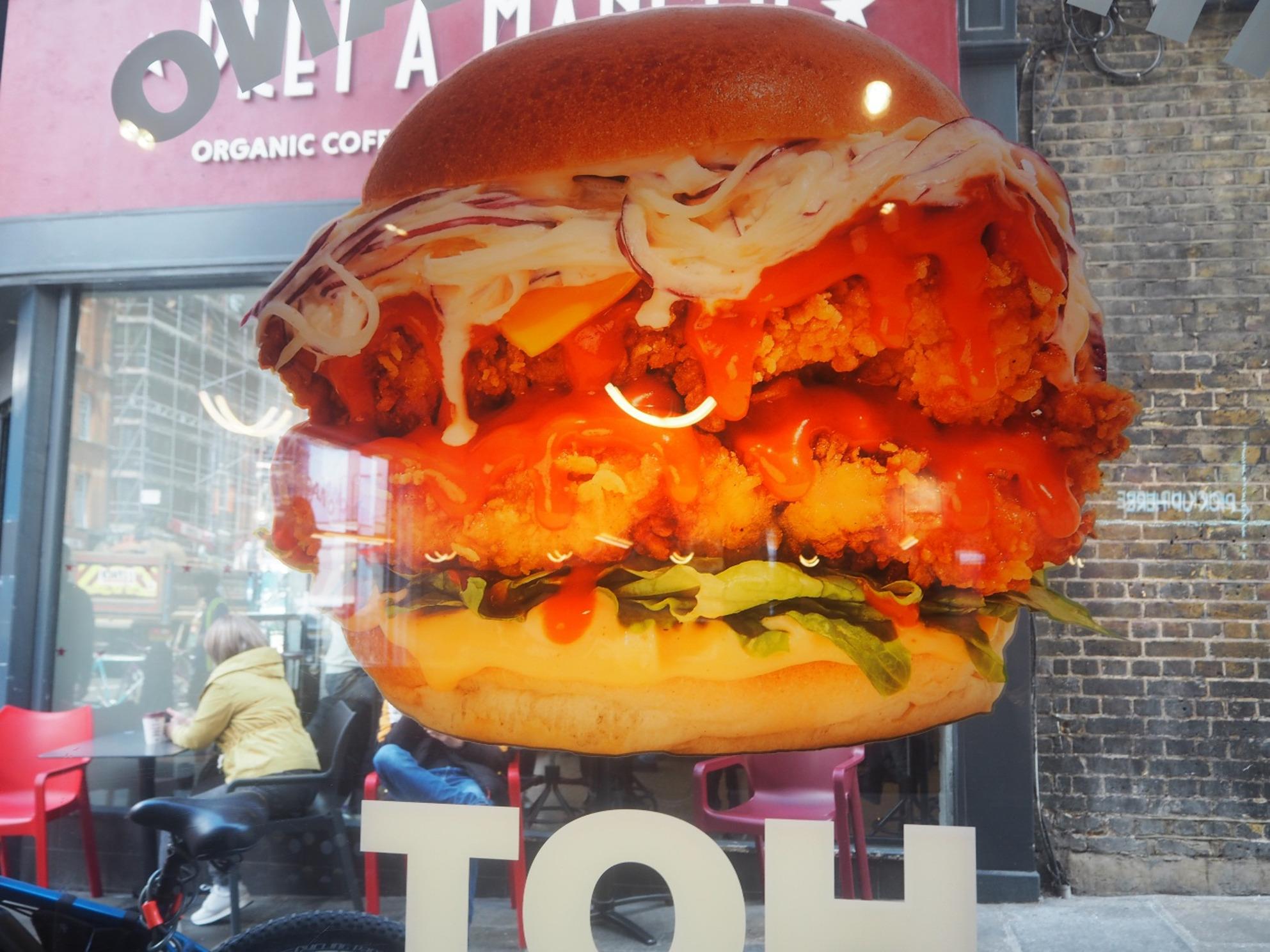
Burger advert. ‘The burger looks so fresh, juicy and delicious but the image is flat against a layered background. It shows how false and disappointing it may be once you see, pay and eat the real thing.’ (Inner-city exhibition)​

**Fig. 6:**
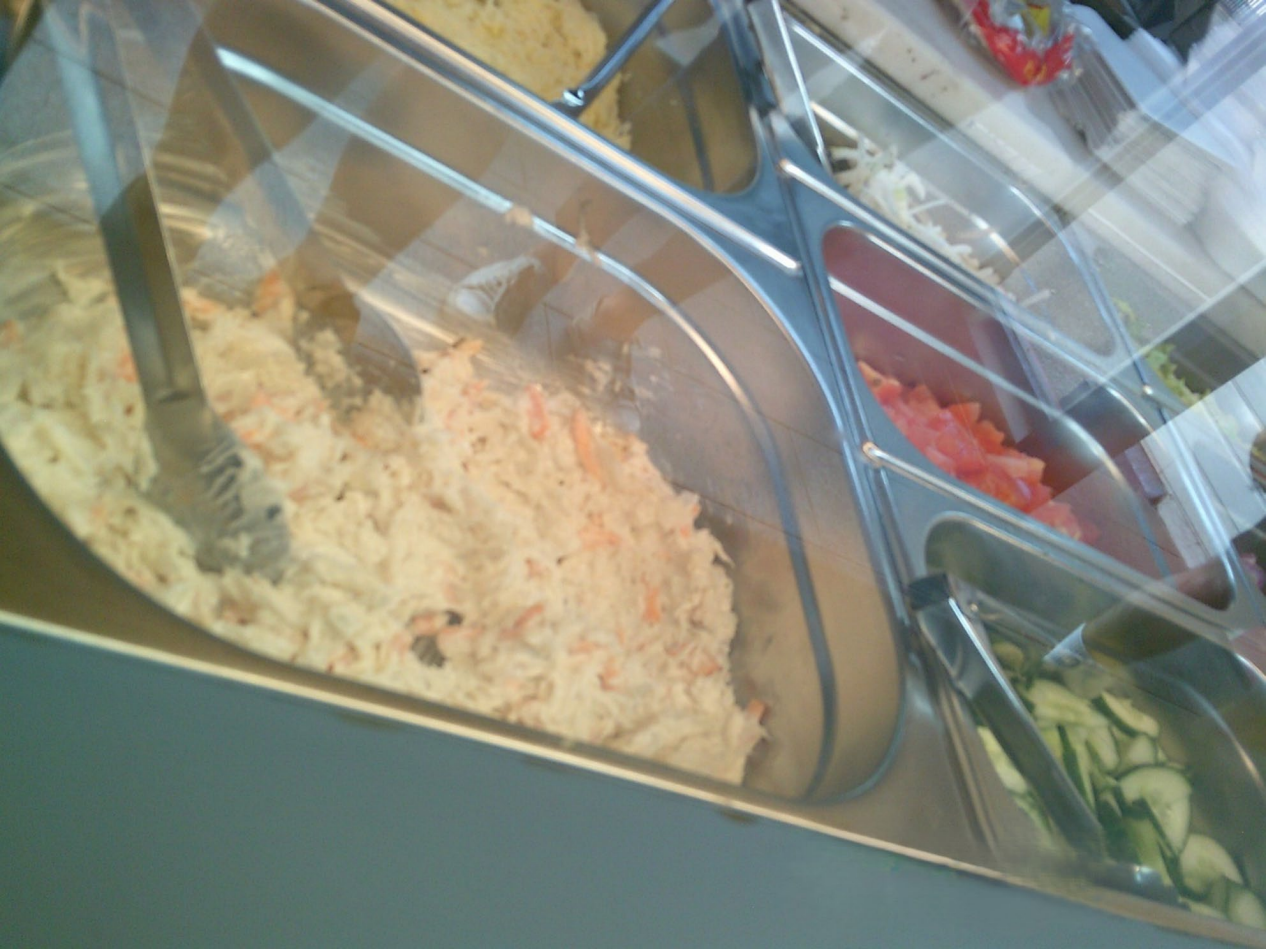
Unhealthy food (Coastal discussion group)

Additionally, misleading marketing tactics were discussed at length, such as the promotion of unhealthy food products and the concepts of ‘health halos’ or ‘greenwashing’, where companies incorrectly and/or indirectly promote their food’s health or environmental credentials to improve the perception of their brand, whilst primarily selling unhealthy products (Figs. 5 and 6). Participants also acknowledged the disadvantage that local and small-scale businesses face when competing against the greater resources of larger food corporations, particularly for advertising and marketing as *“…a lot of the small places don’t have as much money for advertising. So*,* it’s just the big places*,* you have like really big profits*,* they are just advertising because they’ve got the money to spend.” (Inner-city)*.

*‘And it [picture of salad in kebab shop] kind of just*,* like*,* it’s at the front*,* and it draws your attention. So it’s*,* kind of*,* like*,* all colourful and*,* like*,* healthy. It makes you feel like it’s healthier*,* but it’s actually*,* if you look closely*,* you can see everything’s*,* like*,* pretty high in calories.’ (Coastal*,* discussion group referring to* Fig. 6*)*.

### Value placed on the relational aspects of food

To young people, food plays a vital role in bringing people together and fostering connections. When discussing the calorie labelling policy and their local food environments, young people often emphasised the social and cultural significance of food, and they were well aware that food is more than its energy content. They also expressed a strong sense of social responsibility, believing that food should be produced and sold in ways that do not harm society or the environment. This sense of responsibility was reflected in their support for local businesses, preference for ethically produced food, and value placed on food provenance.

#### The role of food in fostering connections

**Fig. 7:**
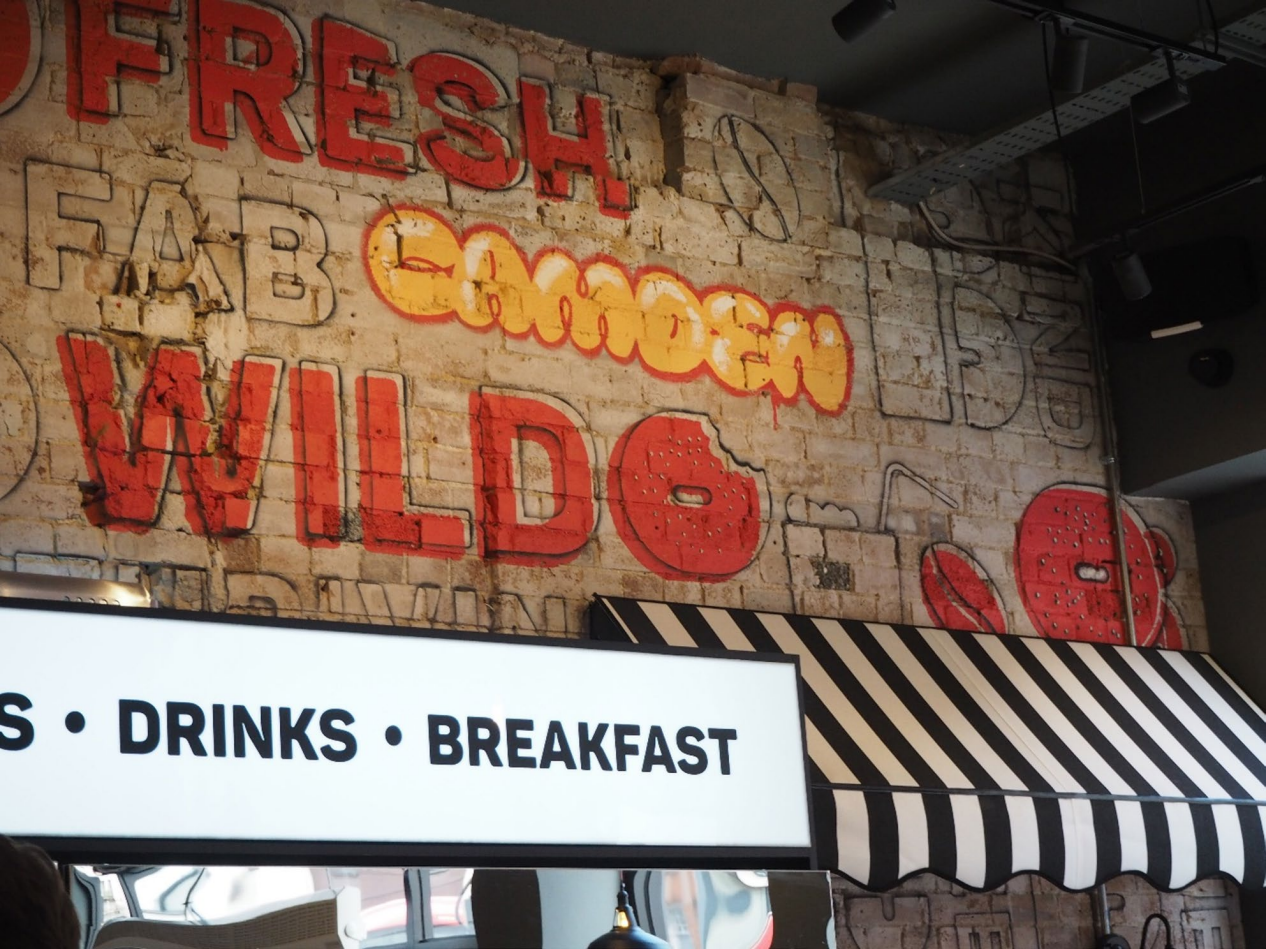
Good vibes. ‘I took this picture because I thought this place had a really nice sitting area where you could just relax and eat. What is important to me when I’m choosing to eat out is the vibe and the way I feel to eat in a place more so the food is offered or the calories that are shown.’ (Inner-city exhibition)

Young people’s accounts of their interactions with their food environments are often centred on the social aspects of eating and the belief that food should be enjoyed rather than solely defined by its calorie content. They described OOH food outlets as places to socialise with friends or celebrate special occasions, with young people drawn to places with a “nice vibe” rather than those that were necessarily healthy or displayed calorie information (Fig. 7).

OOH food outlets could also serve as settings for family rituals. For example, one participant described how their mother would take them and their siblings to a fast-food chain as a reward after grocery shopping.

*‘But*,* urm*,* like every Saturday*,* my mom used to do like shopping and stuff like that. So*,* we would come out*,* we didn’t like that*,* but she likes accompanying us*,* being there. As a reward*,* she’ll give us the McDonald’s. We enjoy it*,* because like we get to order as we like*,* and stuff. But she wanted what’s good for us*,* and that’s why she’s trying to minimize it*,* to like in certain*,* like even like three weeks*,* you know…’ (Inner-city)*.

For some, food was an expression of their identity. They spoke with pride about maintaining a connection with their culture and community through food. Their support for local businesses was closely tied to a demand for authentic food and supporting diverse local communities, as shops and stalls selling traditional food tend to be small and independently owned. They sought comfort by eating at smaller, family-owned outlets and lamented how their local food environments had changed over time, with culturally relevant and smaller eateries declining and being replaced by inauthentic and homogenous offerings sold by big chains.

*‘…like Taco Bell and stuff*,* that’s not authentic Mexican food*,* and we*,* all of the places that are*,* I feel*,* are getting closed down because they’re either in predominantly black areas*,* so then it’s low income*,* and then the Caribbean places are getting closed down*,* and we are like*,* well*,* where am I supposed to eat*,* where*,* when I want to reconnect to my culture?’* (*Inner-city*).

#### From food consumers to food citizens – social responsibility

**Fig. 8:**
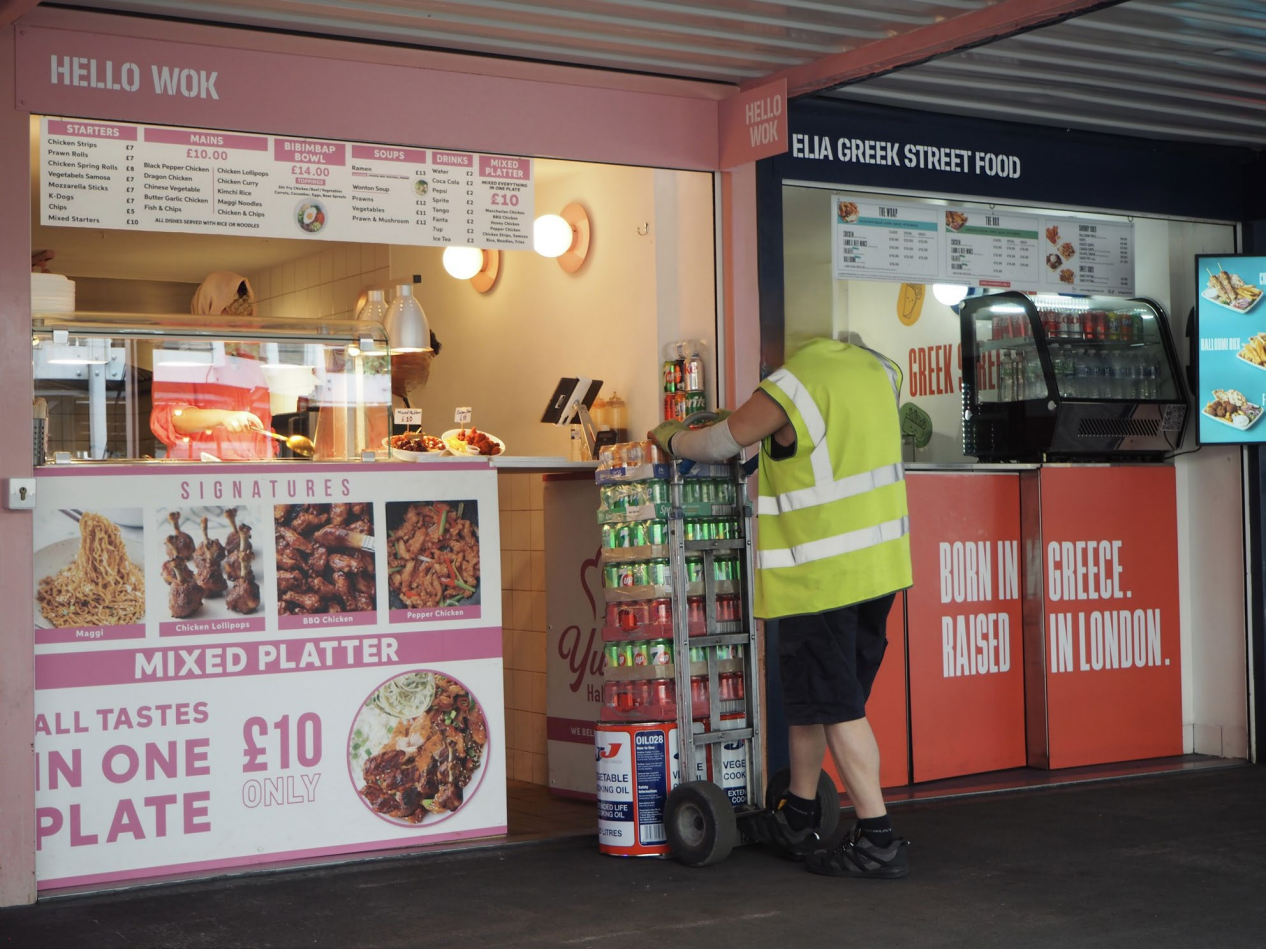
Local businesses. ‘I took this photo to show the difference in buying from a small or family run business. It’s more direct ordering straight from the owners of the business, in comparison to a big corporation, which also makes you feel guilty if you don’t (order)… When I go out to eat, I prefer to support local businesses because my buy is noticed and makes a difference.’ (Inner-city exhibition)

**Fig. 9:**
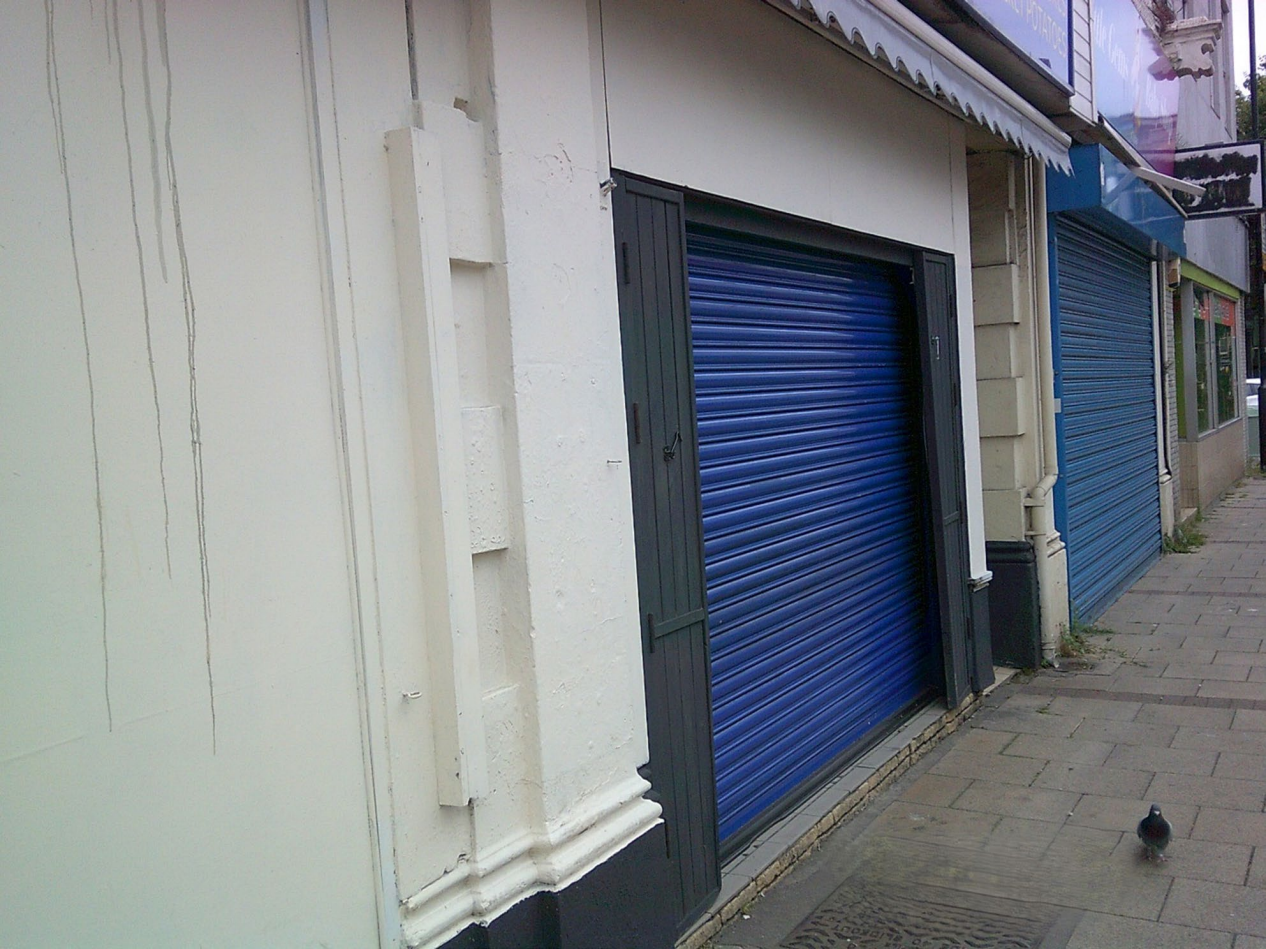
Transnational Corporations. ‘Transnational Corporations are making it harder for small local businesses who often sell healthier and locally sourced food.’ (Coastal exhibition)

For many young people, food choices were guided by a strong sense of community values. They believed food environments should be driven by the health and wellbeing needs of communities, rather than the profits of unknown corporations. Supporting local businesses was seen to be a way to give back to the community and maintain a relationship rooted in mutual care. To them, local businesses were more than commercial spaces; they were integral to maintaining the social fabric of their communities, which contributed to community life. They discussed small, family-run shops that genuinely cared for their customers (Fig. 8) and how reducing the diversity of food shops where they lived might limit their access to healthier or more locally sourced food options (Fig. 9).

**Fig. 10:**
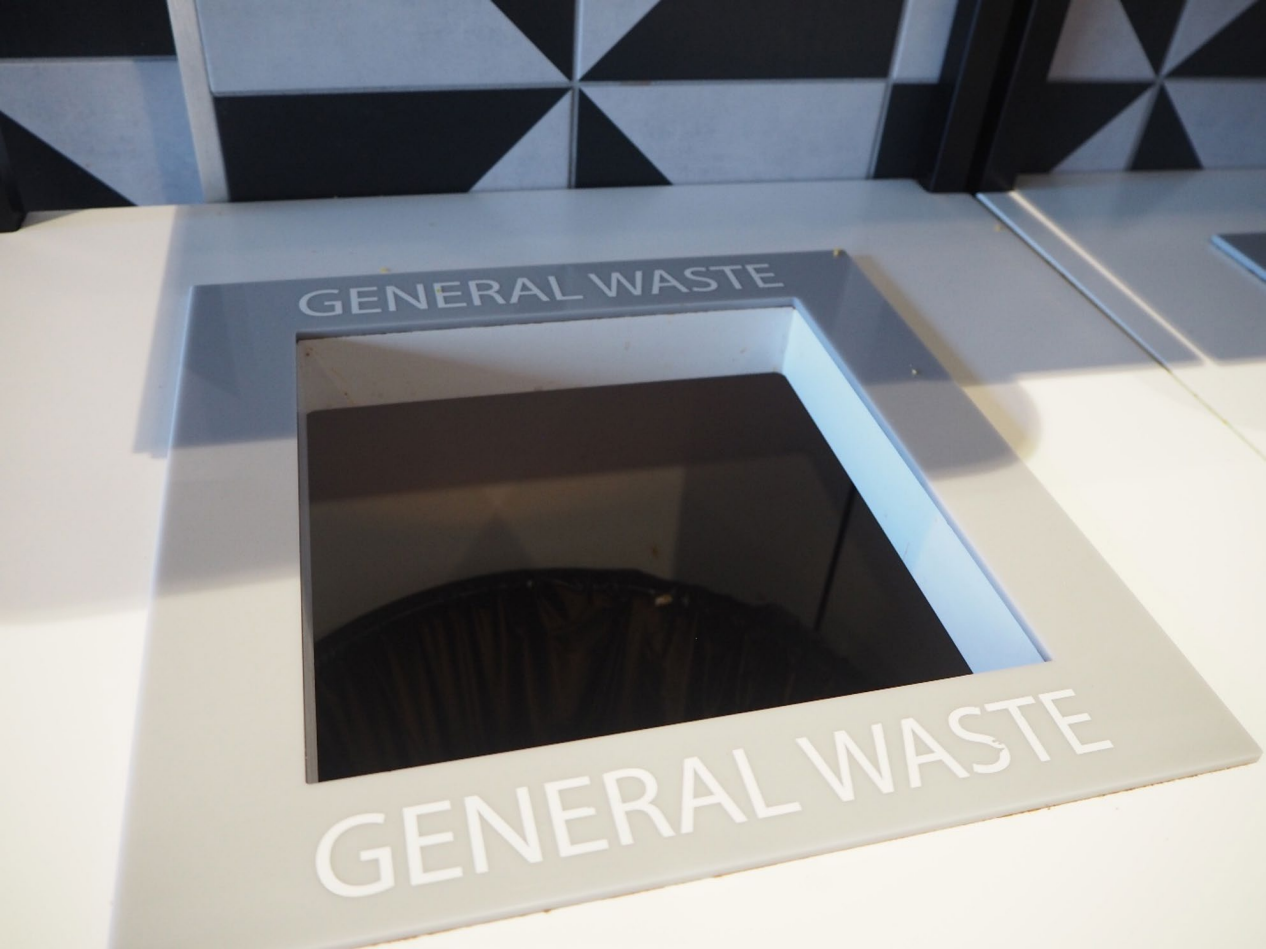
General Waste. ‘I took this photo because at many restaurants people are encouraged to buy bigger meals as they are better value for money however sometimes people cannot finish these unreasonably large portions and they just get thrown in the bin and wasted.​ This waste of product is serious issue as the material used to make the meals have often been grown on deforested land and wasting this food makes it feel like these trees were cut down for nothing. Furthermore, many people in the world are starving and need food but others are just disposing it without thought.’ (Inner-city exhibition)

While the discussions were framed around the calorie labelling policy and their local food environment, young people demonstrated a nuanced understanding of how their food choices intersect with wider issues related to food systems. Their reflections extended beyond health concerns to include both the social and relational aspects of food and the broader environmental impacts of what they eat. Some spoke about the mistreatment of livestock, the exploitation of low-wage labour, and the amount of food waste generated (Fig. 10). Underpinning these reflections was a strong sense of social responsibility and advocacy for greater accountability from businesses regarding how food is produced and sold.

## Discussion

This study explored the views of young people on the calorie labelling policy and their priorities for change in local food environments. Findings suggest the policy did not fully resonate with young people to help them make healthier food choices, or as a mechanism to encourage wider food systems change, such as encouraging food businesses to reduce the energy content of the foods they sell. Participants gave a rich account of the challenges of navigating complex food environments and highlighted a wide range of factors influencing their food choices that were more important to them than calorie information in OOH food outlets. Crucially, participants emphasised the need to address both individual and structural determinants of obesity. They expressed a desire for nutritional information to be relatable and useful in helping them make informed food choices. Additionally, they believed more could be done to improve food environments to meet the health, social, and wellbeing needs of local communities rather than prioritising the profits of food corporations.

### Perceived effectiveness of the calorie labelling policy

Our findings reveal that the calorie labelling policy and current approaches to nutrition education are inadequate in supporting young people in making informed, healthy food choices. Young people generally found calorie information to be of little relevance when making food choices. Instead, they expressed a desire for more holistic, consistent, and coherent information regarding the ‘healthiness’ of foods, as well as their social and environmental impact. They did not consider nutrition education in schools as a helpful source of information. Conversely, they turned to nutrition information online but were still highly sceptical of the credibility of such information on social media. This tension reflects a preference for information that is accessible, relatable, and credible. Previous studies have shown that they seek health information on social media because videos are easy to access and learn from and allow them to engage with presenters who are relatable and can contextualise health knowledge that accounts for young people’s experiences [49, 50]. Further, these findings suggest that nutrition education should encompass the health, social, and environmental sustainability of food systems [51, 52], aligning with longstanding calls from food systems scholars and educators. They also highlighted the importance of critical thinking skills to help young people navigate the overwhelming volume of nutritional information from diverse and often conflicting sources. This is particularly important in today’s world and in the context of widespread mis- and disinformation, especially on social media platforms [50].

While calorie information was not seen as relevant for everyday food decisions, young people recognised that the calorie labelling policy could play a useful role in raising awareness of the energy content of certain items, in particular sugar-sweetened milk and coffee drinks. This observation is timely, given the UK government’s decision to extend the Soft Drinks Industry Levy to include milk-based or milk-substitute drinks with added sugars [53]. This suggests that the calorie labelling policy could complement broader policy efforts to reduce total energy intake from free sugars, which remains twice the recommended level among under-18s [8].

At the same time, the potentially harmful consequences of the calorie labelling policy for young people were acknowledged. Although mental health impacts did not feature prominently in discussions, participants raised them as an important concern. This aligns with previous research involving young adults and adult populations, which shows that experiences with calorie labelling are influenced by body image concerns, and that such exposure can exacerbate eating disorders or mental health symptoms [54]. Our findings support careful consideration and monitoring of unintended consequences of the policy to minimise harm, particularly among vulnerable adolescents. It also underscores the importance of ongoing research examining the UK calorie labelling policy through the lived experiences of adolescents with eating disorders [54].

### Priorities for change in young people’s food environments

Our findings show that young people recognised and supported both structural-level and individual behaviour change interventions for tackling diet-related health inequalities. Participants acknowledged that food environments could be improved, but the calorie labelling policy was not considered a key lever for such change. In contrast, young people more readily discussed the structural determinants of diet and health, such as poverty and commercial interests. Their emphasis on structural solutions is supported by literature and policy responses that recognise the powerful role food environments and commercial actors play in shaping food choices [55–58]. This perspective also aligns with recent studies in the UK on young people’s views of health inequalities, where individual-level interventions were rarely proposed as viable options; instead, more radical, whole-systems change solutions were presented to address health inequalities [59, 60]. Our findings highlight the need for more structural, systems-level interventions that improve food environments, making healthy food choices easier and more accessible for young people.

Participants were aware of how commercial practices shaped their food environments and how this might be widening inequalities by constraining access to healthier choices in OOH food outlets. This was evident in two ways. First, participants recognised the considerable power of commercial entities in determining which food products are available and how accessible or desirable these are [57, 58]. Where nutrient-poor foods are ubiquitously available at low price points, this significantly shapes individual choices and exacerbates diet-related health inequalities [61]. This tension was evident in participants’ attitudes and actions. Although they viewed large chains as profit-driven and selling nutrient-poor foods, they nonetheless purchased from these outlets due to low prices and convenience. This highlights how a lack of affordable and accessible healthier options reinforces reliance on chain outlets, and how food choices are shaped by the interplay of multiple structural factors, including commercial practices, rather than solely by individual preferences or beliefs. Second, they highlighted the decline in independent food outlet diversity and the dominance of large, branded chains on their high streets. This represents a trend seen across the UK following the 2008 financial crisis, which saw a rapid decline of independent shops, largely replaced by chain outlets [62, 63]. This phenomenon is particularly pronounced in areas of high deprivation, in which a higher density of fast-food chains is found in more deprived areas compared to more affluent ones [10], and this has been associated with widening inequalities in obesity rates [64]. Our findings highlight the need for policy and regulatory measures to counter the influence of commercial entities, including strategies to improve access to nutritious, affordable foods in OOH outlets and to reduce the prevalence of nutrient-poor, low-cost food options in disadvantaged areas.

While social and cultural relationships are often overlooked in food policymaking, our study demonstrates the critical role they play in daily food practices. Participants keenly discussed the social and cultural significance of food, including their pride and affinity for culturally authentic food. They also described reciprocal relationships with local food businesses, even when this involved frequenting less healthy hot food takeaways, illustrating the importance of community ties and mutual care. While this may seem to contrast with their recognition of the structural determinants of diet, including the influence of commercial entities, it underscores that social and cultural factors can be equally or more influential in shaping behaviour in certain contexts. This is consistent with evidence that care, social connection, belonging, and identity are key factors influencing food choices [51, 52]. Our findings emphasise that effective interventions for healthy eating should incorporate the social and cultural dimensions of food and leverage existing community connections.

### Young people as engaged and active agents in the food system

Taken together, our findings demonstrate how young people are informed food citizens navigating complex and inequitable food environments. Contrary to the conventional portrayal of young people as passive food consumers with little agency [65], our findings show they saw themselves as active agents in the food system. While they recognised barriers and challenges that restricted their ability to change the food system, they demonstrated an understanding of social responsibility and described actions within their control, including supporting local shops with strong community ties and reducing food waste. These attitudes perhaps reflect the rise in youth activism around issues such as climate change and racism, all of which are rooted in structural inequalities [66, 67]. As the generation most likely to be impacted by future food system transformations, young people’s voices must be included in public discourse and policy decisions around food [30]. Researchers, policymakers, and practitioners can actively support young people’s participation in food-related decision-making through meaningful engagement with youth councils; participatory and inclusive approaches like Photovoice, which embed youth voices and foster dialogue with policymakers; and the involvement of young people’s advisory groups in research.

### Strengths and limitations

A key strength of this study was the meaningful involvement of young people as advisors in the research process (the YPAG). The YPAG not only gained new skills in research, but their involvement made the research relevant and accessible to the study participants, providing deeper insights throughout the project.

Another strength was the use of Photovoice and a community walkabout, which was an accessible way for young people to consider and express their views. We recognised that the scope of this study was necessarily constrained, as it was conducted within the context of a broader evaluation of the calorie labelling policy in England [68]. Unlike traditional participatory research, in which participants often have greater agency to shape the research agenda, this study focused specifically on the policy. Nevertheless, the use of Photovoice offered flexibility, enabling the inclusion of young people’s perspectives on their local food environments and on food labelling more broadly. Furthermore, the exhibitions also promoted dialogue and provided participants and their families the opportunity to present their views directly and be heard by strategic stakeholders.

Qualitative research does not aim to be representative; rather, it seeks to provide rich, in-depth descriptions of a phenomenon, with all perspectives considered valuable. The selection of an inner-city area and a coastal town was intended to capture diverse perspectives, as these regions typically differ in ethnicity profiles and in the drivers of deprivation and health inequalities. Additionally, rather than comparing findings between sites, we interpreted them collectively to identify common insights that may not have been apparent within individual sites. Nonetheless, purposive sampling based on age and area-level deprivation may have limited the diversity of views and experiences captured.

## Conclusion

Young people viewed calorie labelling as irrelevant or even unhelpful for making healthy eating choices and did not see it as vital for changing the local food environment. Instead, they emphasised the need to address structural barriers to healthy eating and to support young people in navigating increasingly complex and inequitable food systems. They advocated for more actions to tackle these barriers alongside interventions aimed at individual behaviour change. Importantly, they demonstrated their role as informed food citizens by exercising social responsibility to shape their food environments. It is vital to amplify their voices in public discourse and support their participation in shaping food policy and governance.

## Supplementary Information

Supplementary Material 1.

Supplementary Material 2.

Supplementary Material 3.

## Data Availability

Requests for access to anonymised data extracts may be considered by the research team, subject to ethical review and a data sharing agreement that ensures participant confidentiality is maintained.
