# Supplementary material for: ‘We should be focusing on why we eat, what we eat and how it makes us feel, not how many calories it has’: a photovoice study exploring young people’s views on the out-of-home calorie labelling policy in England and their priorities for changing the local food environment
Source: BMC Public Health. 2026 Feb 24;26:1056. doi: 10.1186/s12889-026-26716-7 (PMC13036947; doi:10.1186/s12889-026-26716-7)
Supplement: Supplementary file 1 — Supplementary Material 1. [file 12889_2026_26716_MOESM1_ESM.docx]

**
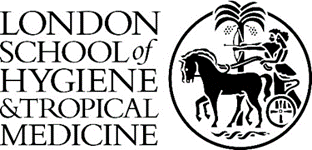
**
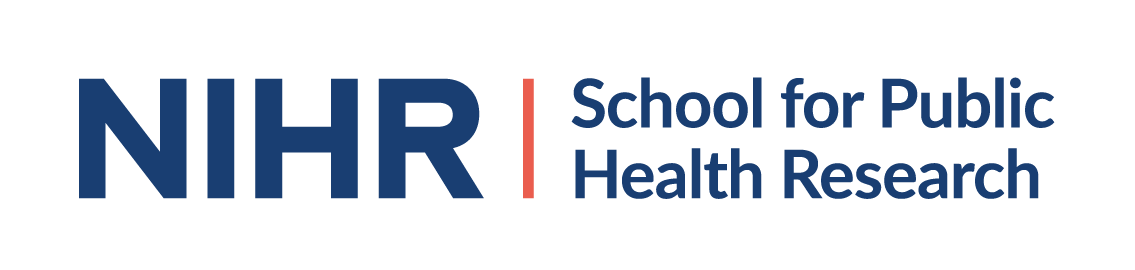

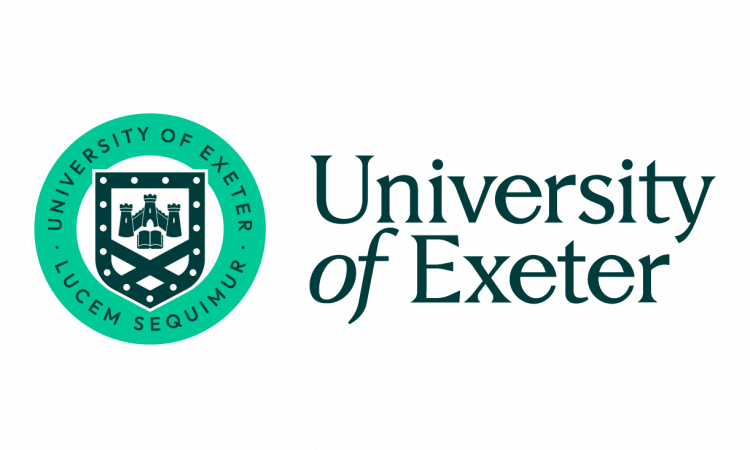


Please sign or initial the sections to indicate your agreement with each statement

This form refers to photographs that you supplied, or photographs that you allowed researchers at the London School of Hygiene and Tropical Medicine and Exeter university to make, as part of the ‘Calorie Labelling Policy Project’ in which participated. All photographs will be securely stored by the research team. As discussed with you, photographs may be shared within the research team to help them in their analyses. We would also like to use some photographs (in electronic or print form), in reports, presentations, publications and photo-exhibitions arising from the project. Please could you sign one of the boxes below to indicate whether or not you are happy for us to do this. We won’t use any photographs outside the research team without your permission.

**Please sign either 1, 2, or 3 below:**

1. I give my consent for these photographs to be reproduced for educational and/or non-commercial purposes, in reports, presentations, publications, websites and exhibitions connected to the Calorie Labelling Policy project.

Signed.............................................................................. date...................................................

**OR**

If you would like to give permission for us to publish some, but not all, of the photos please list the numbers of the photos you will allow us to use:

2. I give my consent for photo numbers........................................................................................ (please specify) to be reproduced (in electronic or print form), for educational and/or non-commercial purposes, in reports, presentations, publications, websites and exhibitions connected to Calorie Labelling Policy project.

Signed............................................................................... date...................................................

**OR**

**3.** I do not wish any of these photographs to be reproduced in connection with the Calorie Labelling Policy project

Signed............................................................................... date...................................................

**Researcher details**

| **Thank you for participating in our project. If you have any queries about this form or about the project or your participation in it, please do not hesitate to contact**  **London/LSHTM:** 15-17 Tavistock Place, London WC1H 9SH **Telephone: 020 927 2177**  **Email: Vanessa Er** [vanessa.er@lshtm.ac.uk](mailto:vanessa.er@lshtm.ac.uk) / Dalya Marks [dalya.marks@lshtm.ac.uk](mailto:dalya.marks@lshtm.ac.uk)  **Exeter:** Camilla Forbes [c.a.mchugh@exeter.ac.uk](mailto:c.a.mchugh@exeter.ac.uk) and Kerry Ann Brown [K.A.Brown@exeter.ac.uk](mailto:K.A.Brown@exeter.ac.uk) |
| --- |
