# Supplementary material for: ‘We should be focusing on why we eat, what we eat and how it makes us feel, not how many calories it has’: a photovoice study exploring young people’s views on the out-of-home calorie labelling policy in England and their priorities for changing the local food environment
Source: BMC Public Health. 2026 Feb 24;26:1056. doi: 10.1186/s12889-026-26716-7 (PMC13036947; doi:10.1186/s12889-026-26716-7)
Supplement: Supplementary file 2 — Supplementary Material 2. [file 12889_2026_26716_MOESM2_ESM.docx]

**Session 1 – plan/topic guide**

The overarching aim of this session is for participants to get to know more about study, the upcoming sessions, as well as each other and the research team. It will also be an opportunity to learn more about how to use the digital cameras and how to take photos.

**Objectives:** ‘Getting to know session’

- Get to know each other
- Get to know the study
- Get to know practical and ethical aspects of photography
- Get to know the camera and develop skills in taking photographs

**Example timings (3 hours including lunch):**

10.30-10.50 – Introductions and ‘getting to know’ activity

10.50-11.05 - Recap of the study and the sessions etc.

11.05-11.25 - Pick and share

11.25-11.45 - Camera and photography tips

11.45-12.30 – Treasure hunt and lunch break

12.30-13:00 - Feedback on treasure hunt

13:00-13.30 - Recap, take home activity, feedback and conclude

**Details of activities sessions:**

*1) Introduction (20 minutes):*

- Researchers to introduce themselves and their role in the session i.e one will run the session, the other will be taking notes and if they need anything there to help etc., also introduce any other staff/adult in the room and their role
- Give an outline of what today’s session will cover
- Icebreaker activity ‘Getting to know each other’:

Name their favourite food, introduce themselves and what they like about the food and what they hope to learn from taking part?

- Agree group ground rule, such as:
  - Respect each other’s opinion
  - Actively listen to each other
  - Don’t interrupt when someone is talking
  - Take it in turns
  - Be polite and kind

NB: Ask participants if there’s anything else they would like to add

*2) The study (15 minutes):*

- Check that everyone has read the PIS and understands generally what the study is about, provide printed copies to anyone who would like one.
- Give a recap of the whole study, its aims and what this part of the study is trying to do and what each session will cover/involve.
- Mention the role of the YPAG’s
- Provide opportunity for questions
- Check all participants have consented to be part of the study (have spare consent forms) and explain withdrawal procedure

*3) Pick and share (20 minutes)*

- Participants pick a photo and share their views on the photo with a partner. Then Dalya explains what the photo is about/why it was taken.
- Describe why we are using photographs, what they can tell us, why they are a useful tool, the language and visual literacy etc

*4) Camera and photography tips (20 minutes)*

- Describe some of the practicalities of taking photos – ie where will the images be stored
- Discuss procedures and ethics for taking photographs of other people and gaining their consent
- Describe the procedure for the loaned cameras (i.e. they are on loan, logged, numbered and will be returned at the end of the project (session 5), explain what happens if lost/stolen etc)
- Describe how to operate the cameras (batteries, SD cards, wrist band etc.)
- Discuss the ‘Four F’s’ (Framing, focus, follow through, flash)

*5) Treasure hunt activity and lunch break (45 minutes)*

- Allow participants to familiarise themselves with the camera and take practice photos.
- Use an activity for participants to gain confidence in photography such as the treasure hunt, ask participants to take a photo of the following:
  - Something that is your favourite colour
  - A pattern
  - A portrait
  - Something natural
  - A detail you think no one else will have noticed

*5) Treasure hunt feedback (30 minutes)*

- Come back together as a group to discuss and look at each other’s photos and how/what they communicate.
- Download 1 photograph from each participant
- Guess what category each photo belongs to. This can be facilitated by using the SHOWED technique:
- S: What do you see here?
- H: What is happening here?
- O: How does this relate to our lives?

*6) Recap and Feedback:*

- Give a quick recap of the session
- Give a brief description of what the next session will focus on
- Give details of the take home activity. Ask participants to take photos on the topic of: Food store that you visit on the way to school/with friends.
- Give participants post-it notes and ask them the following questions:
  - What did you enjoy most about the session today?
  - And what did you enjoy least?
  - What would you take away from today’s session?

**Session 2 – plan/topic guide**

The overarching aim of this session is to explore and understand participants’ awareness, knowledge and opinion of the calorie labelling policy and increase their familiarity with the policy.

**Objectives:**

- Explore and understand YP knowledge/awareness of the calorie labelling policy
- Explore and understand YP opinion of the calorie labelling policy
- Increase YP familiarity with the policy and its aims

**Example timings (3 hours including lunch):**

10.30-10.50 – welcome and recap of the study’s aims and outline of session 2

10.50-11.20 – facilitate a discussion on calorie labels and government policy

11.20-11.30 - short break

11.30-12.15 - facilitate a discussion on the calorie labelling policy

12.15- 12.45- lunch

12.45 -13.15- feedback on Session 1 activity

13.15-13.30- wrap-up, feedback

**Details of activities:**

*1) Welcome and recap of study aims (20 minutes)*

- Give a brief recap of the study aims and offer opportunity to ask questions
- Provide outline of next 3 sessions and answer any questions
- Icebreaker (What special power would you like to have? Why?)

*2) Knowledge and awareness of calorie labels and government policy (30 minutes)*

- Use laminated menu prompts as a visual aid to prompt discussions about calorie labelling
- Possible questions:
- *What do you see here?*
- *Have you seen anything like this – i.e. calorie labels*
- *What does it mean?*
- *Do you know what calories are?*
- *Why do you think the calorie label is there?*
- Move on to calorie labelling policy, using school policy as a conversation starter.
- Possible questions:
- *Has anyone heard of policy?*
- Name a school policy/Give an example of a school policy.
- Why do schools have policies?
- Explain government policies. Then ask about food related policies.*
- *Are you aware of any food policy?*
- *Why do you think we might need this policy?*

***Prompts**: Free school meals, food labels (traffic light), ban/restrictions on ‘junk food’ advertising

*3) Knowledge and awareness of the calorie labelling policy*

- Introduce the calorie labelling policy
- Explore participant’s awareness of the policy.
- Possible questions:
- *Are you aware of the calorie labelling policy?*
- *If yes, what is your experience of it?*
- *Why do you think it was introduced? or What do you think it hopes to do?*
- *What else do you know about it?*

*4) Opinion of the policy*

- Explain what the policy hopes to achieve
- Facilitate a discussion of YPs opinion of the policy
- Again, make sure this discussion is recorded
- Possible questions:
- Do you think this policy will help the government achieve its aim, if no why not?
- What do you think of the aims?
- Are there other ways the government could achieve its aim?
- What do you think are the good things about this policy?
- What do you think are the not so good things about this policy?
- Any other thoughts about the policy?
- Do you think this policy will make people change their food choices? If yes, why? If no, why not?
- Now you know more about the policy will you change how you choose your food in restaurants/takeaways?

*5) Lunch and pinpoint on map*

- Put up a map of the area (to be defined prior to workshop)
- Before participants break for lunch, ask participants to marked up areas/locations of food retailers/stores they frequented.

*6) Feedback on activity after Session 1*

- Make sure the recorder is on and check all are happy for the session to be recorded
- Second researcher to take notes
- Recap on activity
- Participants select one photo from Session 1 take home activity and share with the group why they took the photo/what it means.
- Encourage discussions/reflections

*7)  Wrap up etc.*

- Give a quick recap of what this session has been about
- Give a brief description of what the next session will focus on
- Feedback
- Give participants post-it notes and ask them the following questions:
- What did you enjoy most about the session today?
- And what did you enjoy least?
- What would you take away from today’s session?

**Session 3 – plan/topic guide**

The overarching aim of this session is to conduct a ‘walk about’ session in the local high-street to elicit YP views of the labelling policy and their priorities for change within the local food environment.

**Objectives:** ‘Walk about session’

- Facilitate YP to explore their local food environment on a walkabout
- Elicit YP views of the calorie labelling policy through photography
- Elicit YP views of priorities for change in food availability and access where they live through photography
- Further develop YP camera skills
- Further develop YP knowledge of ethical considerations of photography

**Example timings (3.5 hours including refreshments):**

10.00-10.40 – Introduce the session, safety procedure and reminders of ethical photography

10.40-10.45 – Break

10.45-12.45 – Accompany YP to the chosen local high street/pre-agreed walking route. ‘Walk about’ the local high street, chatting with YP about their impressions/views of the local food environment.

12.45-13.20 – Refreshments and debrief

13.20-13.30 – Recap and conclude

**Details of activities sessions:**

*1) Introduction (45mins):*

- Introduce the session and the aim which is to gain their views on the calorie labelling policy and their local food environment
- Discuss logistics of the walkabout
- Outline/describe the route (share printed-out maps)
- State timings including when to return to the meeting point for recap
- Split into groups (if necessary) and assign a leader
- Outline safety procedures for the walkabout
- Meeting point and contact information if separated from the group
- Inform leader if need to leave or inform leader if someone in the group left/is missing
- Be aware of surroundings when taking photos – e.g. traffic, allow pedestrians to walk past (do not block walkways), other hazards
- Stop taking photographs if being asked to or when YP feel uncomfortable/unsafe to do so.
- Reminder of ethics of taking identifiable photos (person/premise) and what the procedure for this group is.
- Check YP have cameras and that they work. If not, provide a backup or ask YP to use their phone’s camera if they have one.
- Ask for permission if taking photographs of a person (identifiable). Briefly explain what it is for and provide an information sheet for more details. Or refer them to the researchers.
- Respect a person’s right to refusal and be aware of sensitivities around photography.
- Provide 5-10 minute break before departing– toilet, etc.

*2) Walkabout (2 hours)*

- Researchers will accompany YP on pre-agreed walking routes
- Researchers to do a quick head count
- Researchers to monitor for risk and threats, distress/upset
- If YP experience distress/upset, provide the option to leave and ensure they have onward support and report the incident
- Researchers to record what was discussed on the walkabout if feasible and use as possible prompts in the following focus group
- YP will be encouraged to take photos that reflect their view of the calorie food labelling policy and their priorities for change

- Researchers will encourage participants to chat as they ‘walkabout’ to help them express their views:
- What do you think of the food shops around here?

**Potential prompts:** What do you like about the food in your area?  What don’t you like?

- Which shops do you go to? Why?

**Potential prompts:** What type of shops would you like to have in your area?

- Do the shops have what you need?

**Potential prompts:** Are there any foods that you can’t get here? Are they affordable?

- Go into a food outlet that have implemented the calorie labelling policy and look at a menu. If time permitted, go to different types of food outlets (e.g. cafe/restaurant, fast-food outlet)
- What do you notice about the menu?
- How is the information about calories presented?
- What do you think of it (presentation)?
- What information you would like to see on menus or foods

*3) Lunch and Recap: (35 minutes)*

- Allow 10 minutes for YP to get refreshments before a sit-down discussion
- Researchers ask YP to share reflections on the walkabout
- What do you think of the walk?
- Did anything stand out/catch your attention?

*4) Recap and conclude; (10minutes)*

- Give a brief description of what the next session will focus on
- Ask YP to select a couple of photos that they would like to share with the group for Session 4
- Feedback. Give participants post-it notes and ask them the following questions:
- What did you enjoy most about the session today?
- And what did you enjoy least?
- What would you take away from today’s session?

**Session 4 – plan/topic guide**

The overarching aim of this session is to capture YP views of the labelling policy and their priorities for change within the local food environment.

**Objectives:**

- Capture YP views of the calorie labelling policy through focus group discussion of photographs
- Capture YP views of priorities for change in food availability and access where they live through focus group discussion of photographs

**Example timings (4.5 hours including refreshments):**

10.30-10.50 – Welcome and recap of the study’s aims and outline of session 4

10.50-12.20 – Focus group discussion (share and discuss walkabout photo) Part 1

12.20-12.50 - Lunch

12.50-13.20 - Focus group discussion Part 2

13.20-13.30 - Break

13.30-13.50 - Facilitate discussion of any topic/issue of interest that wasn't covered

13.50-14:00 - Recap and conclude

**Details of activities:**

*Focus group*

- Session will be recorded, and notes taken
- Each participant will select up to 2-3 favourite photographs. Participants would have been asked to do this prior to the workshop. Researchers should have them ready to share on screen.
- There will be a facilitated discussion for the participants to explain why they took the photographs and what they mean to them. This will be conducted using the SHOWED technique e.g.,
- S: What do you See here? (prompts: please describe your photograph)
- H: What is really Happening here? (interpretation of what the photograph means)
- O: How does this relate to Our lives/topic (prompts: or to you?)
- E: Why does this problem, situation, or concern Exist?
- D: What can we Do about it? (prompts: what would you change if you could? What would make it easier/harder for you to eat more healthy foods?)

Researchers will also encourage participants to reflect on their walkabout experiences by asking their views on:

- Food menus
- Potential prompts: What do you notice about the menu? How is the information about calories presented? What do you think of it (presentation)?What information you would like to see on menus or foods
- The food outlets in their area (availability)
- Potential prompts: What do you think of the food shops around here? What do you like about the food in your area?  What don’t you like? What type of shops would you like to have in your area?
- Where they buy food/eat out in their area (access)
- Potential prompts: Which shops do you go to? Why?
- Do the shops have what you need (availability and access)
- Potential prompts: Are there any foods that you can’t get here? Are they affordable?

*Facilitate discussion of any topic/issue of interest that wasn’t covered*

Note: Additional time set aside for researcher to ask participants any topic/issue of interest that wasn’t covered in the focus group discussion.

*Recap and conclude*

- Give a quick recap of what this session has been about
- Give a brief description of what the next session will focus on
- Feedback
- Give participants post-it notes and ask them the following questions:
- What did you enjoy most about the session today?
- And what did you enjoy least?
- What would you take away from today’s session?

**Session 5 – plan/topic guide**

The overarching aim of this session is adding captions to the photos from the ‘walk about’ session, to reflect the themes that have emerged from sessions 2-4.

**Objectives:** ‘caption session’

- Provide YP opportunity to understand captioning
- Provide opportunity for YP to caption their photos for display
- Reflect on the themes emerging from session 2-4
- Plan the exhibition

**Example timings (1-2 hours):**

3.30-3.40 – introduce the session and reintroduce the exhibition

3.40-3.50 – introduce the concept of captions

3.50-4.30 – facilitate session regarding captioning YP chosen photos

4.30-4.40 –present emerging themes from session 2-4 and how these link to photos

4.40-5.00- plan exhibition

5.00 – 5.15 conclude/recap, feedback

**Details of activities sessions:**

*1) Introduction:*

- Introduce the session
- Reintroduce the exhibition, explain any arrangements that are already in place

*2) Caption concept*

- Introduce the concept of captions
- If time do an example activity

*3) Captioning chosen photos*

- Ask YP a series of short questions and seeing if their answers would make good captions for each chosen photograph. The questions will involve using the SHOWeD technique:
- *(S); What do they see*
- *(H) How does it make them feel;*
- *(O)How is it related to their own lives;*
- *(W)What opportunity or problem does it represent; and*
- *(D)what do they think would help to change the situation?*
- Notes will be taken of this session

*4) Themes*

- Present the theme summaries from session 2&3
- Facilitate a discussion to sense check the themes and how their chosen images relate to these themes

*5)* *Exhibition planning*

*Facilitate a conversation to involve the group in planning the exhibition*

- *Time and location*
- *Invitees*
- *Space and display*

*6) Recap and Feedback:*

- Camera return?
- Give a quick recap of the session
- Hand out feedback forms?
