# Supplementary material for: ‘We should be focusing on why we eat, what we eat and how it makes us feel, not how many calories it has’: a photovoice study exploring young people’s views on the out-of-home calorie labelling policy in England and their priorities for changing the local food environment
Source: BMC Public Health. 2026 Feb 24;26:1056. doi: 10.1186/s12889-026-26716-7 (PMC13036947; doi:10.1186/s12889-026-26716-7)
Supplement: Supplementary file 3 — Supplementary Material 3. [file 12889_2026_26716_MOESM3_ESM.docx]

**Session 1 – plan/topic guide**

The overarching aim of this session is for participants to get to know more about study, the upcoming sessions, as well as each other and the research team. It will also be an opportunity to learn more about how to use the digital cameras and how to take photos.

**Objectives:** ‘Getting to know session’

- Get to know each other
- Get to know the study
- Get to know practical and ethical aspects of photography
- Get to know the camera and develop skills in taking photographs

**Example timings (3 hours including lunch):**

10.30-10.50 – Introductions and ‘getting to know’ activity

10.50-11.05 - Recap of the study and the sessions etc.

11.05-11.25 - Pick and share

11.25-11.45 - Camera and photography tips

11.45-12.30 – Treasure hunt and lunch break

12.30-13:00 - Feedback on treasure hunt

13:00-13.30 - Recap, take home activity, feedback and conclude

**Details of activities sessions:**

*1) Introduction (20 minutes):*

- Researchers to introduce themselves and their role in the session i.e one will run the session, the other will be taking notes and if they need anything there to help etc., also introduce any other staff/adult in the room and their role
- Give an outline of what today’s session will cover
- Icebreaker activity ‘Getting to know each other’:

Name their favourite food, introduce themselves and what they like about the food and what they hope to learn from taking part?

- Agree group ground rule, such as:
  - Respect each other’s opinion
  - Actively listen to each other
  - Don’t interrupt when someone is talking
  - Take it in turns
  - Be polite and kind

NB: Ask participants if there’s anything else they would like to add

*2) The study (15 minutes):*

- Check that everyone has read the PIS and understands generally what the study is about, provide printed copies to anyone who would like one.
- Give a recap of the whole study, its aims and what this part of the study is trying to do and what each session will cover/involve.
- Mention the role of the YPAG’s
- Provide opportunity for questions
- Check all participants have consented to be part of the study (have spare consent forms) and explain withdrawal procedure

*3) Pick and share (20 minutes)*

- Participants pick a photo and share their views on the photo with a partner. Then Dalya explains what the photo is about/why it was taken.
- Describe why we are using photographs, what they can tell us, why they are a useful tool, the language and visual literacy etc

*4) Camera and photography tips (20 minutes)*

- Describe some of the practicalities of taking photos – ie where will the images be stored
- Discuss procedures and ethics for taking photographs of other people and gaining their consent
- Describe the procedure for the loaned cameras (i.e. they are on loan, logged, numbered and will be returned at the end of the project (session 5), explain what happens if lost/stolen etc)
- Describe how to operate the cameras (batteries, SD cards, wrist band etc.)
- Discuss the ‘Four F’s’ (Framing, focus, follow through, flash)

*5) Treasure hunt activity and lunch break (45 minutes)*

- Allow participants to familiarise themselves with the camera and take practice photos.
- Use an activity for participants to gain confidence in photography such as the treasure hunt, ask participants to take a photo of the following:
  - Something that is your favourite colour
  - A pattern
  - A portrait
  - Something natural
  - A detail you think no one else will have noticed

*5) Treasure hunt feedback (30 minutes)*

- Come back together as a group to discuss and look at each other’s photos and how/what they communicate.
- Download 1 photograph from each participant
- Guess what category each photo belongs to. This can be facilitated by using the SHOWED technique:
- S: What do you see here?
- H: What is happening here?
- O: How does this relate to our lives?

*6) Recap and Feedback:*

- Give a quick recap of the session
- Give a brief description of what the next session will focus on
- Give details of the take home activity. Ask participants to take photos on the topic of: Food store that you visit on the way to school/with friends.
- Give participants post-it notes and ask them the following questions:
  - What did you enjoy most about the session today?
  - And what did you enjoy least?
  - What would you take away from today’s session?
